# Metabolic engineering of *Methylobacterium extorquens* AM1 for 1-butanol production

**DOI:** 10.1186/s13068-014-0156-0

**Published:** 2014-10-21

**Authors:** Bo Hu, Mary E Lidstrom

**Affiliations:** Department of Chemical Engineering, University of Washington, Seattle, WA USA; Department of Microbiology, University of Washington, Seattle, WA 98195-1750 USA

**Keywords:** *Methylobacterium extorquens* AM1, 1-butanol, Ethylmalonyl-CoA pathway

## Abstract

**Background:**

Butanol is a promising next generation fuel and a bulk chemical precursor. Although clostridia are the primary industrial microbes for the fermentative production of 1-butanol, alternative engineered hosts have the potential to generate 1-butanol from alternative carbon feedstocks via synthetic metabolic pathways. *Methylobacterium extorquens* AM1, a facultative methylotrophic α-proteobacterium, is a model system for assessing the possibility of generating products such as 1-butanol from one-carbon and two-carbon feedstocks. Moreover, the core methylotrophic pathways in *M. extorquens* AM1 involve unusual coenzyme A (CoA)-derivative metabolites, such as crotonyl-CoA, which is a precursor for the production of 1-butanol.

**Results:**

In this work, we engineered a modified CoA-dependent pathway in *Methylobacterium extorquens* AM1 to produce 1-butanol. Engineered strains displayed different 1-butanol titers using ethylamine as a substrate. A strain overexpressing *Treponema denticola* trans-enoyl-CoA reductase, *Clostridium acetobutylicum* alcohol dehydrogenase, and native crotonase was able to generate the highest 1-butanol titer (15.2 mg l^−1^). *In vitro* isotopic tracing of metabolic flux and *in vivo* metabolite analysis showed the accumulation of butyryl-CoA, demonstrating the functionality of the synthetic pathway and identifying targets for future improvement.

**Conclusions:**

We demonstrated the feasibility of using metabolic intermediates of the ethylmalonyl-CoA pathway in *M. extorquens* AM1 to generate value-added chemicals, with 1-butanol as the test case. This will not only establish the biotechnological potential of the ethylmalonyl-CoA pathway, but will also introduce *M. extorquens* AM1 as a potential platform to produce value-added chemicals.

## Background

1-butanol has been proposed to be a better alternative to ethanol as a replacement for gasoline because of its many advantages [1]. 1-butanol is less soluble in water than ethanol, reducing the possibility of contamination in groundwater, and this trait also makes it feasible to use liquid-liquid extraction for 1-butanol recovery in industrial fermentation. Unlike ethanol, 1-butanol is compatible with the current petroleum infrastructure and can be blended with gasoline in a high ratio [2]. The energy density of 1-butanol is close to that of gasoline, so one gallon of gasoline blended 50:50 (v/v) with 1-butanol produces a similar mileage to that of pure gasoline [3,4].

Fermentative production of 1-butanol by clostridial species has a long history as the main industrial 1-butanol-producing process [4]. Although recent development of new genetic tools for solventogenic clostridia has led to evolved strains with a combination of desired traits [5,6], industrial fermentation of clostridia still has limitations such as formation of byproducts, spore formation, and low cell density [7]. Therefore, there is growing interest in metabolically engineering alternative hosts for 1-butanol production. Recently, several groups have reported successful reconstruction of the butanol synthesis pathway of clostridia in non-native hosts, including *Escherichia coli* [8], *Saccharomyces cerevisiae* [9], *Pseudomonas putida*, *Bacillus subtilis* [10], and *Lactobacillus brevis* [11]. While starchy substrates or molasses are major carbon feedstocks for fermentative production of 1-butanol in clostridia or heterologous microorganisms, interest is growing in employing novel substrates that are economically competitive with petrochemical synthesis yet at the same time non-competitive with food for human consumption [12]. In the last few years, attention has focused on single carbon compounds such as methane and methanol as future alternative carbon feedstocks due to their relative abundance [13]. However, 1-butanol has not yet been reported to be produced by organisms capable of growing on methane (methanotrophs) or methanol (methylotrophs).

**Figure 1:**
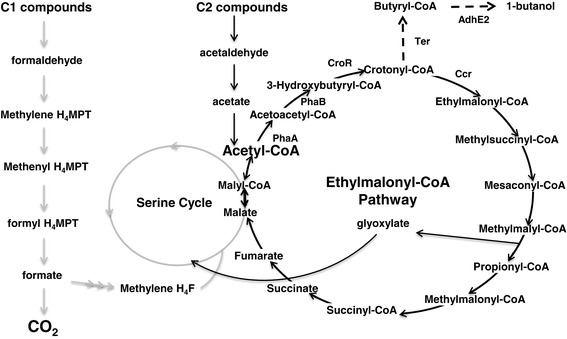
**Ethylmalonyl-CoA pathway in** ***M. extorquens*** **showing intermediates for 1-butanol production.** PhaA, β-ketothiolase; PhaB, acetoacetyl-CoA reductase; CroR, crotonase; Ccr, crotonyl-CoA carboxylase/reductase; Ter, trans-2-enoyl-CoA reductase; AdhE2, bifunctional aldehyde/alcohol dehydrogenase. Gray lines represent methylotrophic pathways used exclusively in C1 assimilation. Dashed lines represent the heterologous pathway for 1-butanol production.

*Methylobacterium extorquens* AM1 is a facultative methylotrophic α-proteobacterium capable of using both one-carbon (C1) compounds as well as multi-carbon compounds as sole carbon and energy sources. The potential practical significance of *M. extorquens* AM1 in biotechnology, such as in the biosynthesis of amino acids and single-cell proteins and the bioconversion of methanol into products with economic value, has brought it into prominence since the 1960s [14]. As the most well-understood methylotroph, *M. extorquens* AM1 is a potential platform for converting methanol to biofuels, building on the elucidation of pathways involved in C1 and C2 metabolism and development of tools for metabolic engineering. The ethylmalonyl-CoA pathway (EMC pathway, Figure 1), a central metabolic pathway in *M. extorquens* AM1, converts acetyl-CoA to glyoxylate for reincorporation into the serine cycle during C1 assimilation and replenishes metabolites that either leave the cycle for biosynthetic purposes or are consumed by the tricarboxylic acid (TCA) cycle during C2 assimilation [15,16]. The EMC pathway involves unusual metabolites that are of interest for the production of valuable compounds, such as crotonyl-CoA, a key precursor of the 1-butanol biosynthesis pathway in clostridia (Figure 1). Moreover, it has been shown that significant metabolic flux occurs through the EMC pathway during growth of *M. extorquens* AM1 on either C1 or C2 compounds, generating a stable supply of crotonyl-CoA as a direct precursor for 1-butanol production [17]. Therefore, in this work we describe the engineering of a modified CoA-dependent pathway in *M. extorquens* AM1 to produce 1-butanol.

## Results and discussion

### Construction of 1-butanol biosynthesis pathway

The essential enzymes for 1-butanol production are outlined in Figure 1, three of which (PhaA, PhaB, and CroR) are already present in the EMC pathway of *M. extorquens* AM1. In addition to these existing enzymes, heterologous enzymes were introduced to convert crotonyl-CoA to 1-butanol. These include an enzyme catalyzing the reduction of crotonyl-CoA to butyryl-CoA (Bcd and EtfAB from *Clostridium acetobutylicum*, or Ter from *Treponema denticola*) and an alcohol dehydrogenase from *Clostridium acetobutylicum* that converts butyryl-CoA to 1-butanol [18,19]. Two alcohol dehydrogenases are present in *Clostridium acetobutylicum*: AdhE1 and AdhE2 [20]. Although engineered strains overexpressing either of the homologs produced a similar level of 1-butanol [10], AdhE2 was thought to be the more active homolog [21] and thus was used in this study for the reduction of butyryl-CoA. The enzyme-coding genes were expressed in a polycistronic manner driven by promoters developed for overexpression in *M. extorquens* AM1. Four promoters were tested, ranging from high to low expression, including those for *mxaF*, *lac* [22], and two chosen based on microarray data [23–25].

Engineered strains were tested for 1-butanol production using methanol or ethylamine as the carbon source. Notably, no methanol-grown strains produced detectable 1-butanol. We hypothesized that the methanol dehydrogenase in *M. extorquens* AM1, a pyrroloquinoline quinone (PQQ)-dependent periplasmic alcohol dehydrogenase that can interconvert C1-C4 alcohols and aldehydes, may interfere with net conversion of butyraldehyde to 1-butanol by catalyzing the reverse reaction in the periplasm [26]. Therefore, we also tested 1-butanol production from a *mxaF* mutant growing on methylamine, which does not contain significant methanol dehydrogenase activity [27]. However, no 1-butanol was detected, suggesting that endogenous butanol dehydrogenase activity may not be an important factor causing low 1-butanol titer. An alternative explanation is that the glyoxylate generated by the EMC pathway is essential for growth on methanol [28]. Tests for 1-butanol production from cells grown on methanol plus glyoxylate at a concentration known to rescue mutants in the EMC pathway for growth on methanol [29] did not result in detectable 1-butanol production, suggesting that lack of glyoxylate production was not a limiting factor.

**Figure 2:**
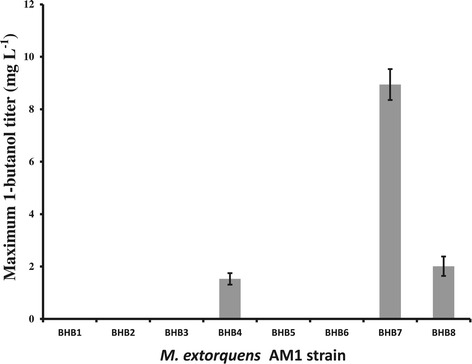
**Maximum 1-butanol titers of engineered strains.** Cells were grown in minimal medium with 20 mM ethylamine in shake flasks for 4 days. Error bars represent standard deviation of triplicate experiments.

Although the constructs tested were unable to produce 1-butanol during growth on methanol, three of the strains (BHB4, BHB7, and BHB8) produce detectable levels of 1-butanol during growth on ethylamine (Table 1), with BHB7 showing the highest titer of 8.9 mg l^−1^ (Figure 2). The titer is comparable with that of early reported organisms such as *S. cerevisiae* (2.5 mg l^−1^, [9]) and cyanobacteria (14.5 mg l^−1^, [30]), but comparison of our strain to the native 1-butanol producers *Clostridium* (about 15 g L^−1^) or the engineered *E. coli* strains (about 15 g L^−1^) demonstrates the need for significant additional 1-butanol titer improvement [31,32]. Growth on C2 compounds is significantly different from growth on C1 compounds, which likely underlies the difference in 1-butanol production. During growth on C2 compounds, fewer steps are required for converting C2 compounds into acetyl-CoA. It has been suggested that the NADH pool is critical to drive 1-butanol synthesis due to the large NADH consumption by the 1-butanol production pathway [31]. Although neither of the NADH-linked formate dehydrogenases is essential for cell growth [33], a stoichiometric model suggests that biomass synthesis is limited by reducing power rather than ATP during growth on C1 compounds, but not on C2 compounds [16,34]. Moreover, during growth on two-carbon compounds, the level of NADH is different than that on methanol [16]. One or more of these differences may be the reason why none of these constructs accumulated detectable amounts of 1-butanol during growth on methanol.

**Table 1:** ***M. extorquens*** **strains and plasmids used in this study**

**Strain**	**Plasmid/Genotype** *	**Reference**
**Wild type**	Rif derivative	27
**BHB1**	pCM66 (Plac:: bcd&etf-adhE2)	This study
**BHB2**	pCM80 (PmxaF:: bcd&etf-adhE2)	This study
**BHB3**	pAP775 (Pmeta1_3616 :: bcd&etf-adhE2)	This study
**BHB4**	pCM80 (PmaxF:: adhE2-ter)	This study
**BHB5**	pCM66 (Plac:: adhE2-ter)	This study
**BHB6**	pAP776 (Pmeta1_002:: adhE2-ter)	This study
**BHB7**	pAP775 (Pmeta1_3616 :: adhE2-ter)	This study
**BHB8**	pAP775 (Pmeta1_3616 :: ter-adhE2)	This study
**BHB9**	pHC61 (Pmtac:: croR:: Pmeta1_3616 :: adhE2-ter)	This study
**Plasmids**	**Description**	**Reference**
**pCM66**	*M. extorquens* expression vector (Plac, Km^R^)	25
**pCM80**	*M. extorquens* expression vector (PmxaF, Tc^R^)	25
**pAP775**	*M. extorquens* expression vector (Pmeta1_3616, Tc^R^)	E. Skovran
**pAP776**	*M. extorquens* expression vector (Pmeta1_002, Tc^R^)	E. Skovran
**pHC61**	*M. extorquens* expression vector (Pmtac, Km^R^)	47

*Promoter strength: PmxaF > Pmeta1_3616 > Pmeta1_002 > Plac.

The three strains that generated detectable 1-butanol in ethylamine-grown cells, BHB4, BHB7, and BHB8, all contained Ter instead of the Bcd and EtfAB cluster. This is possibly due to the oxygen sensitivity and instability of the Bcd and EtfAB complex [18,35], since *M. extorquens* AM1 is an aerobic methylotroph that does not grow under anoxic conditions. The strain that generates the highest 1-butanol titer (BHB7) expresses the heterologous genes from a promoter of intermediate strength (Pmeta1_3616, [23]), suggesting that tuning of expression levels is critical for proper functioning of the 1-butanol production pathway.

### Enzyme activities

Activities of the heterologous enzymes of the 1-butanol pathway were measured in extracts of cells grown on ethylamine. Both butyraldehyde dehydrogenase activity and butanol dehydrogenase activity of AdhE2 were detected above the background activity present in wild-type *M. extorquens* AM1 (Table 2). Attempts to measure a combined Bcd and EtfAB activity were not successful using crude extract from cells that expressed both *bcd* and *etfAB*. The lack of detectable activity may be due to the high oxygen sensitivity of the Bcd and EtfAB assay, as in previous studies that have demonstrated the difficulties of obtaining reliable data for these activities in other engineered microbes [18]. This result is in keeping with the observations above that 1-butanol was not produced by these strains. Ter activities were measured in crude extracts of the engineered strains, and their levels were comparable to the levels of Ccr in extracts of wild-type *M. extorquens* AM1 [36]. Since the native enzyme Ccr competes with Ter for crotonyl-CoA, both the velocity and the Km are important parameters. The reported Km value for Ter with respect to crotonyl-CoA is 2.7 μM, which is 100 times lower than that of the native Ccr (400 μM), and the Km value of a recombinant Ter in *E. coli* with respect to crotonyl-CoA is yet five times lower than that of Ccr [19,37,38]. Therefore, Ter likely generates sufficient metabolic driving force toward the 1-butanol synthesis pathway, supported by the results showing that the Ter-containing strains generate detectable 1-butanol.

**Table 2:** **Enzymatic activities of Ter and AdhE2 in** ***M. extorquens*** **AM1 strains**

**Enzyme**	**Strain**					
**(nmol/min/mg)**	**wild type**	**BHB4**	**BHB5**	**BHB6**	**BHB7**	**BHB8**
**Ter**	9.5 ± 3.3	59.1 ± 19.8	41.5 ± 13.6	52.1 ± 12.2	62 ± 5.61	89.6 ± 22.3
**AdhE2** ^*^	17.5 ± 1.4	21.8 ± 5.4	24.3 ± 3.2	28.2 ± 11.8	31.3 ± 4.8	18.6 ± 2.5
**AdhE2** ^**#**^	1.3 ± 0.4	7.5 ± 2.2	7.4 ± 1.2	6.9 ± 1.8	11.9 ± 0.6	1.6 ± 0.7

*Butanol dehydrogenase activities.

# Butyraldehyde dehydrogenase activities.

### *In vitro* isotopic tracing through reactions in the 1-butanol pathway

**Figure 3:**
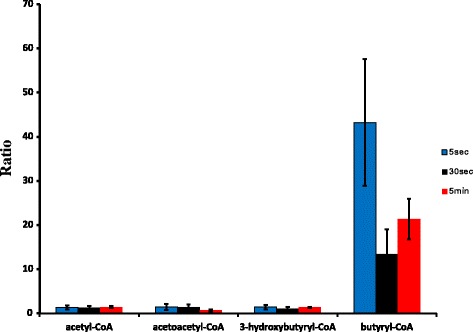
***In vitro*** ^**13**^ **C-based metabolic flux analysis of wild-type and BHB7 strain.** Results are ratios of peak volumes in BHB7 cell extracts compared to wild type. The extracts of ethylamine-grown cells were fed with ^13^C-labeled acetyl-CoA and radiolabeled metabolites were measured at different reaction times: 5 s (blue), 30 s (black), and 5 min (red).

In order to assess overall function of the entire 1-butanol synthetic pathway, assays were carried out using cell extracts of ethylamine-grown wild-type and BHB7 strains incubated with ^13^C-labeled acetyl-CoA, NADPH, and NADH, measuring the accumulation of labeled intermediates over time. A set of parallel experiments were conducted in which reactions were incubated for 5 s, 30 s, and 5 min before quenching and mass spectrometry analysis. Through measurements of the ^13^C isotopomer labeling patterns, we identified four of the five predicted labeled intermediates (all except for crotonyl-CoA) in the 1-butanol-producing pathway (Figure 3).

Labeled acetyl-CoA, acetoacetyl-CoA, and 3-hydroxybutyryl-CoA were detected in similar amounts for the wild type and the BHB7 strain during the time course. Crotonyl-CoA was not detected, even though the precursor to crotonyl-CoA, 3-hydroxybutyryl-CoA, and the downstream metabolite butyryl-CoA were both detected. Previous studies reported the difficulties of quantifying crotonyl-CoA *in vivo* because of the small pool size [15,28]. Small amounts of butyryl-CoA were detected in the assays of the wild type, but the amount was 13- to 43-fold higher in the BHB7 strain. This result is consistent with a previous report that a small amount of butyryl-CoA is detected in cellular extracts of *M. extorquens* AM1 grown on ethylamine [39]. The pathway for butyryl-CoA synthesis in wild-type *M. extorquens* AM1 is not known. It could be a side product of the native reductive carboxylation reaction to form ethylmalonyl-CoA, or it could be generated from isobutyryl-CoA via valine synthesis, or from metabolism of even-numbered fatty acids [37,40]. However, regardless of the source, the rate of butyryl-CoA synthesis in the wild type is very low. The significant increase of labeled butyryl-CoA in strain BHB7 is consistent with the enzymatic function of Ter *in vitro* and shows that carbon flux to butyryl-CoA occurred via the 1-butanol synthetic pathway. In addition, accumulation of butyryl-CoA suggests that increasing the efficiency of AdhE2 might improve the system.

### *In vivo* intermediates of the 1-butanol pathway

**Figure 4:**
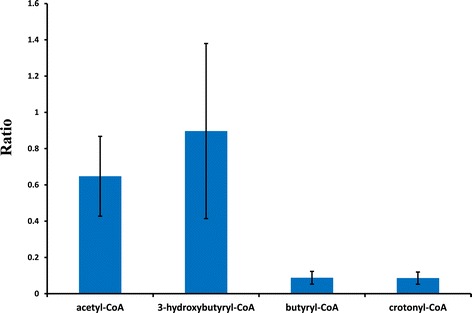
**Comparison of** ***in vivo*** **1-butanol pathway intermediates in wild type and strain BHB7.** Samples were collected from ethylamine-grown cells at mid-exponential phase. Results are ratios of peak volumes in BHB7 samples compared to wild type.

In order to identify potential bottlenecks of the 1-butanol pathway, we investigated *in vivo* intermediates by LC-MS. In these samples, four of the five intermediates were detected in cell extracts of the wild-type and BHB7 strains, all except for acetoacetyl-CoA, which either has a small pool size or is not stable in sample pretreatment. The pool sizes from BHB7 of acetyl-CoA and 3-hydroxybutyryl-CoA do not have significant differences compared to the wild type, suggesting that upstream metabolic fluxes were not significantly affected by the introduction of the 1-butanol pathway (Figure 4). However, the pools of both crotonyl-CoA and butyryl-CoA are more than 10-fold lower in strain BHB7 than in the wild-type strain, presumably due to the depletion of crotonyl-CoA by Ter in the BHB7 strain. The result is expected, since the accumulated butyryl-CoA could not be incorporated by central metabolic pathways in the wild-type strain but can be removed by AdhE2 for 1-butanol synthesis in strain BHB7. These results suggest that an insufficient supply of crotonyl-CoA is a bottleneck for 1-butanol production, indicating this step as a target for further strain improvement.

### Optimization of strain for 1-butanol production

One of the strategies available to adjust the crotonyl-CoA supply is to decrease crotonyl-CoA flux through the EMC pathway. Ccr competes with Ter for crotonyl-CoA directly, but Ccr is essential for growth of *M. extorquens* in both C1 and C2 substrates [41]. Previously, we constructed a CcrR mutant which has half the Ccr activity of the wild-type strain, but maintains the ability to grow on methanol and ethylamine, albeit at a lower growth rate [42]. The constructed plasmid that was successful in strain BHB7 was introduced into this CcrR mutant, which was tested for 1-butanol production on C1 and C2 compounds. However, this strain did not produce 1-butanol on methanol, and a decreased amount of 1-butanol was detected from cells grown on ethylamine compared to the wild type, likely because the CcrR mutant grows more slowly on ethylamine compared to the wild type (data not shown).

**Figure 5:**
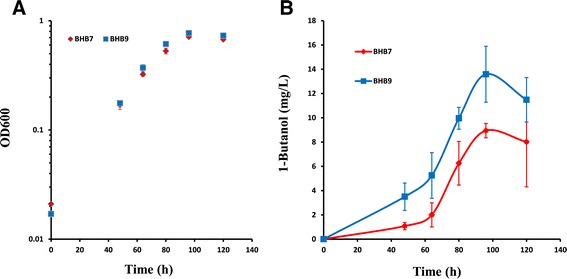
**Effect of CroR overexpression on 1-butanol production. A)** Growth of strain BHB7 (red) and BHB9 (blue). **B)** 1-butanol production in culture of strain BHB7 (red) and BHB9 (blue). All strains were inoculated in medium containing ethylamine (20 mM). Error bars represent standard deviation of triplicate experiments.

An alternative approach to increase the total crotonyl-CoA supply is to overexpress crotonase. The gene encoding crotonase in *M. extorquens* AM1 (*croR*) was cloned under a modified *tac* promoter (CCACACATTATACGAGCCGATGATTAATTGTCAACAGCTCA TTTCAGATTTCTT), which was then assembled with the 1-butanol operon (Pmeta1_3616::*adhE2*::*ter*) in BHB7 to create a new strain, BHB9. A threefold increase in CroR activity was found in the extract of BHB9 compared to BHB7 (0.14 U mg^−1^ protein versus 0.05 U mg^−1^ protein, respectively). When growing on ethylamine, this strain produced 50% more 1-butanol (maximum 1-butanol titer = 13.6 mg l^−1^) and no growth defect compared to BHB7 (Figure 5). These results suggest that higher flux via CroR contributes to the increased production of 1-butanol. The 1-butanol mainly accumulated in the exponential phase.

### 1-butanol tolerance of selected mutants

**Figure 6:**
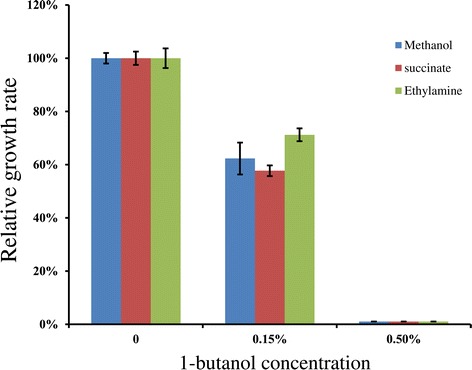
**Growth rates in butanol relative to medium without butanol of wild-type** ***M. extorquens*** **AM1.** Cells were grown on one of the following substrates: methanol (blue), succinate (red), or ethylamine (green). Triplicate experiments were performed for each individual measurement.

1-butanol toxicity to microorganisms is one of the important factors that limit industrial production potential of this next generation biofuel. Therefore, the growth rates of *M. extorquens* AM1 wild type in the presence of 1-butanol were investigated (Figure 6). The growth rate was calculated from growth curves monitored with different carbon sources. The results showed that 1-butanol is toxic to *M. extorquens* AM1 at 0.15% 1-butanol. In the presence of 0.15% butanol, the relative growth rates on methanol, ethylamine, and succinate were 62%, 58%, and 72%, respectively, compared to those for the medium without butanol. However, *M. extorquens* AM1 cannot tolerate and grow in 0.5% or higher butanol on any of these three substrates. This result suggests that further strain development for improved 1-butanol tolerance is necessary for *M. extorquens* AM1.

## Conclusions

In this work we reported metabolic engineering of the ethylmalonyl-CoA pathway in *M. extorquens* AM1 for 1-butanol production. The 1-butanol pathway contains part of the native ethylmalonyl-CoA pathway, a trans-enoyl-CoA reductase from *Treponema denticola*, and alcohol dehydrogenase from *Clostridium acetobutylicum*. The engineered strains demonstrated various maximum 1-butanol titers from cells grown on ethylamine with the highest titer of 8.94 mg l^−1^, which was further improved to 13.6 mg l^−1^ by overexpressing native crotonase. Although further strain optimization is required to make this system industrially relevant, metabolic engineering precedents exist that have resulted in similar magnitudes of increase [43,44]. *In vitro* assays suggested that the enzymatic reaction catalyzed by AdhE2 is a rate-limiting step in 1-butanol synthesis. It has been shown in cyanobacteria that substitution of AdhE2 with oxygen-insensitive butyryl-CoA reductase improves butanol production [45]. Since *M. extorquens* AM1 is an obligate aerobe [46], a similar strategy combined with other efforts such as increasing the reducing power and acetyl-CoA supply can be harnessed to improve 1-butanol production in the future. This study demonstrates that metabolic intermediates such as crotonyl-CoA can be used as substrates of a heterologous engineered pathway to produce value-added chemicals, setting up the first example to develop *M. extorquens* AM1 into a platform for production of biofuels.

## Methods

### Reagents

All chemicals including metabolite standards were purchased from Sigma-Aldrich (St. Louis, MO). Restriction enzymes, Phusion DNA polymerase, ligases, and the Gibson Assembly Master Mix kits were supplied by New England Biolabs (Ipswich, MA). Media components were purchased from commercial resources. The BCA kit for protein measurement was purchased from Thermo Scientific (Waltham, MA).

### Strains, medium, and growth conditions

The strains used in this study are listed in Table 1. *Escherichia coli* strains Top 10 and S17-1 were cultivated at 37°C in Luria-Bertani medium. *M. extorquens* AM1 was routinely cultured in the minimal medium described previously [16] with one of the following substrates: succinate (20 mM), methanol (125 mM), or ethylamine (20 mM). For triparental or biparental matings between *E. coli* and *M. extorquens* AM1, Difco nutrient broth supplemented with Difco BiTek agar (1.5% [wt/vol]) was used. Antibiotics were supplied at concentrations as follows: tetracycline (Tet) 20 μg ml^−1^, kanamycin (Km), 50 μg ml^−1^, ampicillin (Amp), 100 μg ml^−1^, and rifamycin (Rf), 50 μg ml^−1^. 1 mM of glyoxylate was used for tests for 1-butanol production from cells grown on methanol plus glyoxylate.

Growth curves and 1-butanol production assessments were carried out in biological triplicates. Tested *M. extorquens* AM1 strains were subcultured (0.5 ml) from tubes into 50 ml of minimal medium in 250-ml flasks containing the appropriate carbon source, then inoculated at 30°C on shakers at 200 rpm.

To assess the growth rates in the presence of 1-butanol, cultures of *M. extorquens* AM1 wild type were grown to middle exponential phase in 5 ml minimal medium at 30°C. 0.5 ml of each culture was distributed into 50 ml fresh medium in 250-ml screw-capped flasks containing appropriate amounts of 1-butanol, and the growth was followed over time.

### DNA manipulations

The protein sequences of butyryl-CoA dehydrogenase with an affiliated electron transfer flavoprotein (Bcd and EtfAB), alcohol dehydrogenase (AdhE2), and NADH-dependent crotonyl-CoA reductase (Ter) were retrieved from GenBank with the following accession numbers: Bcd and EtfAB, [GenBank:U17110.1], AdhE2, [GenBank:AF321779.1], Ter, [GenBank:AE017248.1]. Genes coding for these enzymes were synthesized into the vector pUC57 (Genescript, NJ, USA) with codon usage optimized for expression in *M. extorquens* AM1 in which the codon usage frequency was calculated by counting codon frequency in a list of ORFs associated with the 129 highly expressed genes in both methanol-grown and succinate-grown *M. extorquens* AM1 cells. The gene encoding crotonase (*croR*) was cloned from *M. extorquens* AM1 genomic DNA. Standard restriction enzyme digestion and ligation techniques were used to construct plasmids except for the one used to create strain BHB9, which was constructed via Gibson Assembly. Genes were PCR amplified with Phusion polymerase and assembled into the *Xba*I-*Bam*HI and *Kpn*I-*Eco*RI restriction sites of plasmids with different promoter regions. Four promoters were tested, one high (*mxaF* promoter of pCM80; [22]), one low (*lac* promoter of pCM62; [25]), and two in between chosen based on published microarray data results (Pmeta1_3616 promoter of pAP775 and Pmeta1_002 promoter of pAP776; [23]). The expression vectors pAP775 and pAP776 were designed and constructed by replacing the *lac* promoter with the putative promoter region upstream of Gene meta1_3616 and Gene meta1_002, respectively. The relative expression of the promoters was PmxaF> > Pmeta1_3616 > Pmeta1_002 > Plac, based on relative microarray expression data. For plasmid construction of strain BHB9, the *croR* fragment was first assembled with a pHC61 vector fragment (under a *tac* promoter [47]), and the new construct was used as a vector fragment to be assembled with the region of Pmeta1_3616::*adh*E2::*ter*.

### Enzyme assays

*M. extorquens* AM1 cells were harvested at mid-exponential phase and then resuspended in the appropriate buffer as described below for each assay. Crude cell extracts were obtained by passing the cells through a French pressure cell at 1.2 × 10^8^ Pa and clarification by 15 min centrifugation at 15,000× g at 4°C. Protein concentrations were determined by the BCA assay using bovine serum albumin as a standard according to the instructions of the supplier. All assays were conducted at 25°C.

### Trans-2-enoyl-CoA reductase (Ter) assay

A standard spectrophotometric assay for Ter activity was performed as described by monitoring the oxidation of NADH at 340 nm [48]. The assay mixture contained 0.5 mM crotonyl-CoA and 0.2 mM NADH in 0.1 M potassium phosphate buffer (pH 7.5). The reaction was initiated by the addition of cell extracts.

### Butanol dehydrogenase (AdhE2) assay

The butanol dehydrogenase activity was measured in the reverse direction as described by monitoring the increase in absorbance at 340 nm [49]. The assay mixture contained 19.6 mM butanol, 0.39 mM NAD+, and 78.5 mM semicarbazide hydrochloride in 68.8 mM Tris–HCl, pH 7.8. The reaction was initiated by the addition of cell extracts.

### Butyraldehyde dehydrogenase (AdhE2) assay

The butyraldehyde dehydrogenase activity was measured by monitoring the oxidation of NADH at 340 nm. The assay mixture contained 200 μM butyryl-CoA, 0.4 mM NADH, and 5 mM dithiothreitol in 0.1 M potassium phosphate buffer (pH 7.5). The reaction was initiated by the addition of butyryl-CoA.

### Crotonase (CroR) assay

The CroR assay was conducted by monitoring the decrease of absorbance at 263 nm, corresponding to hydration of the double bond in crotonyl-CoA. The assay mixture contained 0.15 mM crotonyl-CoA in 0.1 M Tris–HCl buffer, pH 7.6. The reaction was initiated by the addition of crotonyl-CoA.

### 1-butanol quantification

10 ml of culture samples were centrifuged for 10 min at 5,000 rpm. 2 ml ethyl acetate was added to the supernatant. After the addition of 50 mg l^−1^ isobutanol as an internal standard, the mixture was vortexed for 1 min and then centrifuged for 10 min at 5,000 rpm to separate the aqueous phase and ethyl acetate. The recovered ethyl acetate was analyzed by an HP 6890 gas chromatograph equipped with a Model 19091 s-433 HP-5MS column (Agilent) and a single quadrupole mass spectrometer. Helium with 7.64 psi inlet pressure was used as the carrier gas. 1-μl aliquots of the samples were analyzed with the following program: Set initial temperature at 50°C, ramped to 90°C at 10°C/min, ramped to 300°C at 45°C/min, maintained at 300°C for 1 min. A standard curve was derived from measurements of 1-butanol aqueous solutions (2 mg l^−1^, 5 mg l^−1^, 10 mg l^−1^,25 mg l^−1^, 50 mg l^−1^). The ion source temperature was set to 250°C. Mass spectra were collected at m/z 41 and m/z 56 with a 2.2-min solvent delay. The peaks were analyzed using Agilent ChemStation software.

### CoA derivative analysis

For *in vitro* demonstration of the synthetic pathway, cell extracts were prepared in the same way as for enzyme assays. The reaction mixture contained 0.7 mM ^13^C-labeled acetyl-CoA, 0.5 mM NADPH, 0.23 mM NADH, and 0.1 mM Tris–HCl buffer, pH 7.5. The reaction was initiated by the addition of cell extracts and quenched by 3× volume acetonitrile at 10 s, 30 s, or 5 min. The mixture was centrifuged for 10 min at 14,000 rpm after incubation in a −20°C freezer for 1 h. The supernatant was diluted with double-distilled water (ddH_2_O) to 10% acetonitrile and frozen in liquid nitrogen before lyophilization. Each lyophilized sample was dissolved in 50 μl ddH_2_O for LC-MS analysis.

For *in vivo* metabolite measurements, the samples for the determination of extracellular metabolites were collected from cultures when the cells had reached an OD600 of 0.5. 10 ml of cell culture was filtered using a Millipore membrane (0.22 μm), and the membranes were then washed with 3 to 5 ml ice-cold quench buffer (21 mM ammonium formate, 0.17% (v:v) formic acid, and 25% (v:v) ethanol). The cells were broken by passing them through a French pressure cell at 1.2 × 10^8^ Pa twice. The effluent was diluted by ddH_2_O to the total volume of 30 ml. The mixture was then lyophilized to obtain LC-MS samples which were dissolved in 50 μl ddH_2_O .

Both *in vitro* and *in vivo* metabolites were analyzed by a Waters Xevo LC-MS system consisting of an Acquity UPLC system and a Xevo triple quadrupole mass spectrometer (Milford, MA). The LC conditions for the CSH-C18 column (130 Å, 1.7 μm, 2.1 mm × 100 mm) were as follows: mobile phase A consisted of 25 mM amonium acetate, 2% acetic acid (v:v), 1% formic acid (v:v) in water; mobile phase B consisted of 2% acetic acid (v:v), 1% formic acid (v:v) in acetonitrile. Initial A = 100%, set A = 80% at 2.5 min, A = 55% at 5 min, A = 5% at 6 min, A = 100% at 7 min, A = 100% at 8 min. The flow rate was 0.3 ml min^−1^. The MS was operated in the method described before [39] with minor modifications. All data were analyzed using the MassLynx QuanLynx Application Manager software.

## Abbreviations

AdhE1: bifunctional aldehyde/alcohol dehydrogenase
AdhE2: bifunctional aldehyde/alcohol dehydrogenase
ATP: adenosine triphosphate
BCA: bicinchoninic acid assay
Bcd and EtfAB: butyryl-CoA dehydrogenase and electron transferring flavoprotein
Ccr: crotonyl-CoA carboxylase/reductase
CroR: crotonase
EMC: ethylmalonyl-CoA
NADH: nicotinamide adenine dinucleotide
NADPH: nicotinamide adenine dinucleotide phosphate
ORF: open reading frame
PhaA: β-ketothiolase
PhaB: acetoacetyl-CoA reductase
PQQ: pyrroloquinoline quinone
TCA: tricarboxylic acid
Ter: trans-2-enoyl-CoA reductase
